# Experimental–theoretical study of laccase as a detoxifier of aflatoxins

**DOI:** 10.1038/s41598-023-27519-1

**Published:** 2023-01-17

**Authors:** Marco Zaccaria, William Dawson, Darius Russel Kish, Massimo Reverberi, Maria Carmela Bonaccorsi di Patti, Marek Domin, Viviana Cristiglio, Bun Chan, Luca Dellafiora, Frank Gabel, Takahito Nakajima, Luigi Genovese, Babak Momeni

**Affiliations:** 1 Department of Biology, Boston College, Chestnut Hill, MA 02467 USA; 2 RIKEN Center for Computational Science, Kobe, 6500047 Japan; 3 Department of Environmental and Evolutionary Biology, “Sapienza” University of Rome, 00185 Rome, Italy; 4 Department of Biochemical Sciences “Alessandro Rossi Fanelli”, “Sapienza” University of Rome, 00185 Rome, Italy; 5 Department of Chemistry, Boston College, Chestnut Hill, MA 02467 USA; 6 Institut Laue-Langevin, 38042 Grenoble, France; 7 Graduate School of Engineering, Nagasaki University, Nagasaki, 8528521 Japan; 8 Department of Food and Drug, University of Parma, 43124 Parma, Italy; 9 CEA/CNRS/IBS, University Grenoble Alpes, 38044 Grenoble, France; 10 CEA/INAC-MEM/L-Sim, University Grenoble Alpes, 38044 Grenoble, France

**Keywords:** Molecular modelling, Protein design, Molecular engineering

## Abstract

We investigate laccase-mediated detoxification of aflatoxins, fungal carcinogenic food contaminants. Our experimental comparison between two aflatoxins with similar structures (AFB_1_ and AFG_2_) shows significant differences in laccase-mediated detoxification. A multi-scale modeling approach (Docking, Molecular Dynamics, and Density Functional Theory) identifies the highly substrate-specific changes required to improve laccase detoxifying performance. We employ a large-scale density functional theory-based approach, involving more than 7000 atoms, to identify the amino acid residues that determine the affinity of laccase for aflatoxins. From this study we conclude: (1) AFB_1_ is more challenging to degrade, to the point of complete degradation stalling; (2) AFG_2_ is easier to degrade by laccase due to its lack of side products and favorable binding dynamics; and (3) ample opportunities to optimize laccase for aflatoxin degradation exist, especially via mutations leading to π–π stacking. This study identifies a way to optimize laccase for aflatoxin bioremediation and, more generally, contributes to the research efforts aimed at rational enzyme optimization.

## Introduction

Aflatoxins are dangerous fungal secondary metabolites that regularly contaminate crops such as maize, rice, wheat, and peanuts^1^. Aflatoxins are produced by the fungal genus Aspergillus and are among the most carcinogenic natural pollutants^2^. Aflatoxin contamination is a major food safety concern. Physical and chemical detoxification strategies exist, but they can negatively impact food quality and be costly, unreliable, or unsafe^3,4^. In the effort to develop safer alternatives, food recovery through environmentally friendly enzymes has been proposed^5^. To this end, laccase was identified as a good candidate^6–8^.

Laccase is an enzyme of general interest in biotechnology^9,10^. It is a monomeric multicopper oxidase catalyzing one electron oxidations coupled with full reduction of molecular oxygen to water. The active site consists of three copper binding sites with different spectroscopic and functional properties. Type 1 blue copper is the electron acceptor from the substrate; the trinuclear cluster formed by type 2 copper and binuclear type 3 copper is the site of oxygen binding and reduction^11^. Laccase is taxonomically ubiquitous^12^ and functionally versatile: its broad substrate tolerance makes it relevant to industrial applications^9^. Across natural variants, fungal laccases have the highest redox potential (E°), up to 800 mV, at the type 1 copper^12^. Several existing reports have already identified bacterial and fungal laccases which interact with aflatoxins^6,13–17^; however, even the most active isoforms lack time/cost efficiency to satisfy the current demands of aflatoxin detoxification in the food and feed industry. Previous research has focused on optimizing laccase, for different functions, through rational design or directed evolution^18–20^. In the specific context of aflatoxin degradation, molecular docking has provided mechanistic insights^21^, 3D structure analysis of different isoforms assessed interaction with aflatoxins^7^, and mutational analysis explored beneficial changes^22^.

In this work, we combine experimental and computational approaches to pave the way to a rational optimization of laccase as an aflatoxin bioremediator with a focus on aflatoxin B_1_ (AFB_1_), the most carcinogenic congener. We employ laccase from *Trametes versicolor* (TV), a fungal species whose ecological niche is tailored around laccase-mediated lignin degradation^23^. We perform an in-depth analysis of the detoxification of the main target molecule, AFB_1_, by TV laccase to identify the mechanisms behind reaction bottlenecks. Our data also include experiments on an AFB_1_ congener, aflatoxin G_2_ (AFG_2_). By highlighting the remarkable differences in laccase’s activity on AFB_1_ and AFG_2_, despite their structural similarity, we imply that affinity improvement cannot be achieved by specializing the enzyme towards a general category of compounds (e.g. hydrocarbons, aromatic nonphenolic structures, or even aflatoxins as a category). We therefore perform an extensive, high detail quantum mechanical (QM) characterization on the entire TV laccase structure bound to AFB_1_ and AFG_2_, including about 7000 atoms. We predict specific single amino acid residues to be sub-optimal for aflatoxin degradation, and propose the related structural changes to address reaction bottlenecks.

## Results

We first construct a preliminary phenomenological model based on in-vitro laccase activity on aflatoxins. The model highlights two main points: (i) laccase’s efficacy against aflatoxin is not limited by the redox potential of its active site, rather by poor affinity for aflatoxin as a substrate; (ii) AFB_1_, unlike AFG_2_, deviates from the established Michaelis–Menten kinetics characteristic of laccase activity^24^. We then perform a theoretical analysis of the two aflatoxins which reveals the origin of these differences. Finally, we computationally analyze the full laccase-aflatoxin systems to understand which residues contribute to binding, and how.

### Laccase is a more effective detoxifier of AFG_2_ than it is of AFB_1_

In the chemical structure of aflatoxins, the lactone ring is responsible both for the toxicity^25^ and the natural fluorescence of the molecule. As a result, aflatoxin concentration and toxicity can be fluorimetrically assayed. In this work, we will define detoxification as a reaction that breaks the lactone ring in the aromatic structure of aflatoxin, leading to loss of natural fluorescence and toxicity. This assay can be used for both AFB_1_ and AFG_2_ (see “Methods” section).

**Figure 1:**
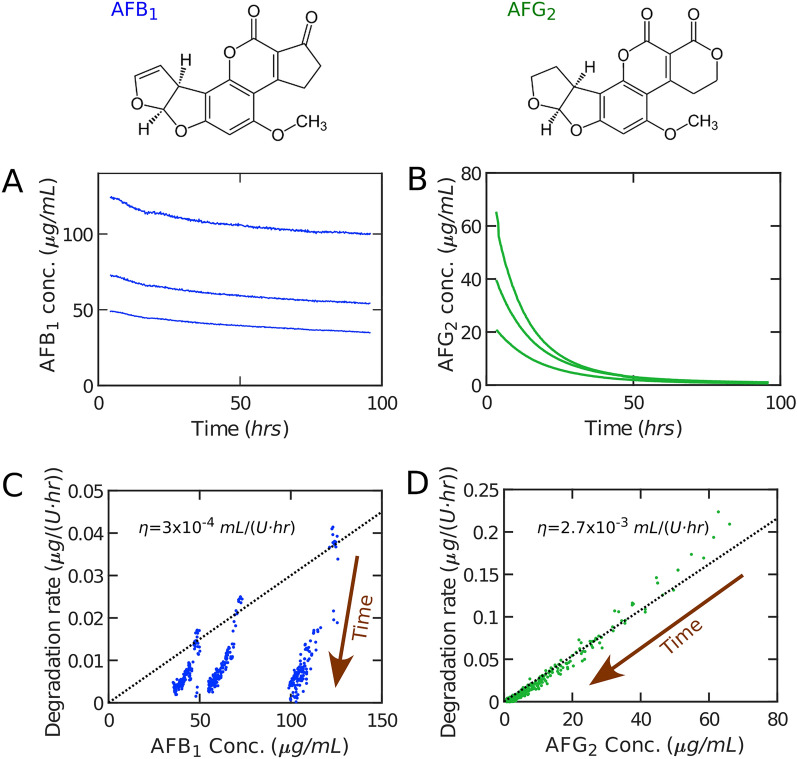
The detoxification of AFB_1_ and AFG_2_ by laccase highlights the difference in detoxification efficiencies even between aflatoxins with similar structure. Different initial aflatoxin concentrations were employed and are represented for AFB_1_ (**A**) and AFG_2_ (**B**). Each curve is the average of 3 replicates. A subset of points from (**A,B**) is randomly selected and represented in (**C,D**) to calculate the local normalized detoxification rates $\left(\frac{-1}{\left[L\right]}\frac{d\left[T\right]}{dt}\right)$. Here, $\left[T\right]$ is the toxin concentration and $\left[L\right]$ is the laccase concentration. Detoxification efficiency $\left(\eta \stackrel{\scriptscriptstyle\mathrm{def}}{=}\frac{-1}{\left[L\right]\left[T\right]}\frac{\Delta \left[T\right]}{\Delta t}\right)$ of AFB_1_ (**C**) is almost an order of magnitude lower than that of and AFG_2_ (**D**) at comparable concentrations. Dotted lines in (**C,D**) illustrate the prediction of the model. Direction of time is represented in (**C,D**) to highlight the decrease in toxin concentration as a result of detoxification. Laccase concentration: 25 U/mL.

We assessed the detoxification activity of TV laccase at different initial AFB_1_ and AFG_2_ concentrations. The fluorimetric assay highlights two distinct detoxification trends for AFB_1_ versus AFG_2_. The AFB_1_ fluorescence readout follows a decreasing trend that, after about 10 h, changes into a slower trend. Overall, detoxification over 96 h amounts to roughly 12% of the original quantity of the toxin (Fig. 1A). AFG_2_ detoxification, in contrast, displays a consistent trend, leading to completion within 96 h (Fig. 1B). Importantly, in the absence of laccase, little detoxification of AFB_1_ and AFG_2_ was observed (Fig. S1).

### Laccase has higher affinity and detoxification rate for AFG_2_ over AFB_1_

To infer the enzymatic activity of laccase against aflatoxins, we assume that detoxification by laccase follows Michaelis–Menten kinetics. We then fit a phenomenological model to our experimental data (see “Methods” section). To test if the toxin concentration is much lower than the Michaelis–Menten constant (${K}_{m}\gg \left[T\right]$), in the experimental data we define the detoxification efficiency as $\eta \stackrel{\scriptscriptstyle\mathrm{def}}{=}\frac{-1}{\left[L\right]\left[T\right]}\frac{\Delta \left[T\right]}{\Delta t}$ (in $\mathrm{mL}/(U\cdot \mathrm{h})$) where $\Delta \left[T\right]$ is the change in the toxin concentration in a small time-step $\Delta t$. Since $\eta =\frac{\rho }{[T]}$ appears to be constant in early degradation (i.e. a linear trend in Fig. 1C,D), we confirm that ${K}_{m}\gg \left[T\right]$ is a valid approximation. Calculating the detoxification kinetics from Eq. (3), and using the value of $\eta$ estimated from experimental data, the model accurately approximates the measured kinetics in the case of AFG_2_ throughout the experimental time (Fig. 1D), further confirming that this model is suitable for representing aflatoxin detoxification by laccase. However, AFB_1_ adheres to the Michaelis–Menten kinetics only for a short time before entering a slower, non-Michaelis–Menten-like detoxification dynamic (Fig. 1C). Thus, compared to AFG_2,_ and other known substrates of laccase, AFB_1_ shows an uncharacteristic trend.

The finding that, at relevant concentrations of the toxin, we get ${K}_{m}\gg \left[T\right]$ can be interpreted as relatively poor activity by laccase for degrading the toxin. We consider the association and enzymatic activity in the standard view^26^:

1 $$L + T\underset{{k_{ - 1} }}{\overset{{k_{1} }}{\rightleftharpoons}}LT\mathop{\longrightarrow}\limits^{{k_{2} }}L + D,$$

where *D* is the detoxified toxin, and ${K}_{m}=\frac{{k}_{2}+{k}_{-1}}{{k}_{1}}\gg \left[T\right]$ means ${k}_{2}+{k}_{-1}\gg {k}_{1}\left[T\right]$. This can be interpreted as low affinity of the enzyme for the aflatoxin, AFB_1_ and AFG_2_ alike, as the rate of association is much smaller than the rates of degradation/dissociation. This low affinity suggests that laccase is naturally not well-adapted to detoxify aflatoxin. As a matter of fact, it has been reported^27^ that under optimized conditions (0.1 M citrate buffer pH 4.5, 20% DMSO 35 °C, TV laccase 30 U/mL) *K*_*m*_ for AFB_1_ was 0.28 mM and the degradation rate with 80 μg/mL AFB_1_ was *k*_*L*_ = 0.89 μg/(U·day). This corresponds to a detoxification efficiency of $\eta =4\times {10}^{-4}$ mL/(U·h) which is comparable to our results reported in Fig. 1C.

### Gas phase modeling and LC–MS reveal intrinsic differences between AFB_1_ and AFG_2_

**Figure 2:**
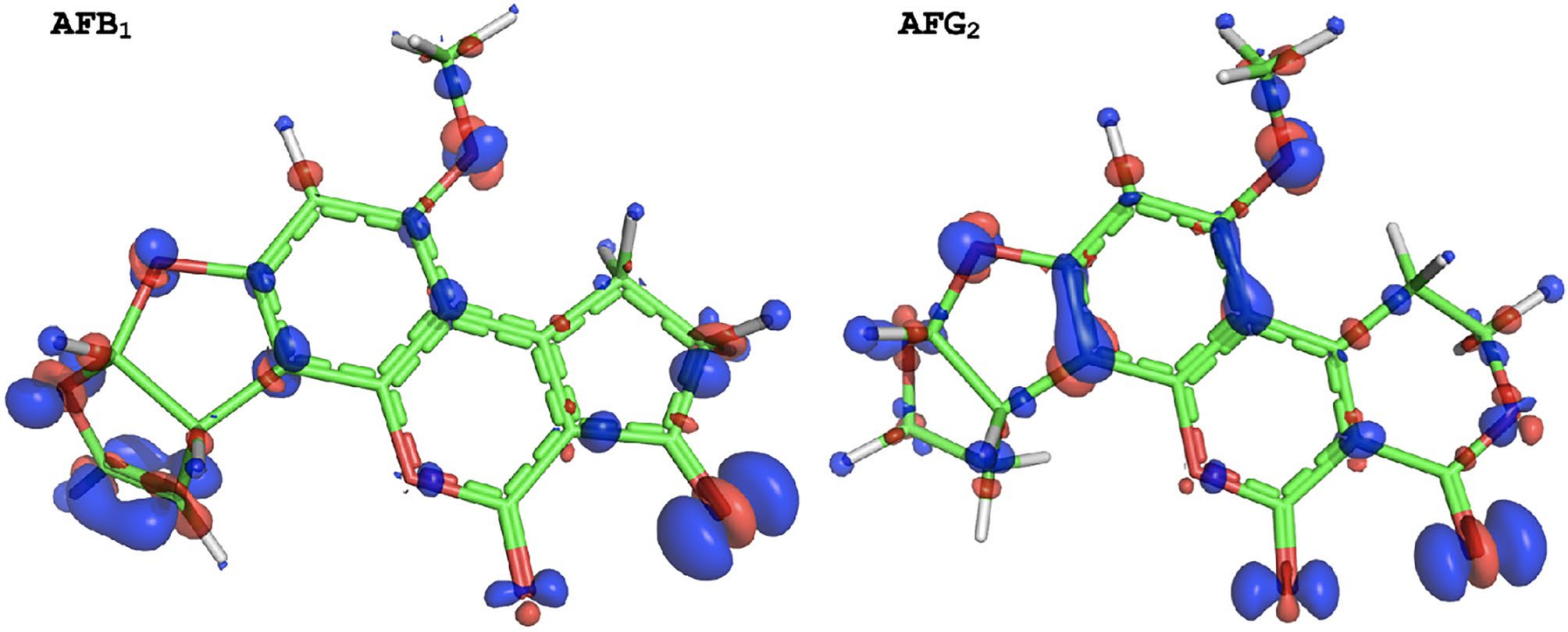
Isosurfaces of the Fukui functions of AFB_1_ and AFG_2_ in the gas phase indicate the sites prone to oxidation. Fukui isosurfaces: red (−) and blue (+). Isosurface level of ± 0.003 Å^−3^.

To understand the differences observed between AFB_1_ and AFG_2_, we investigated the properties of these molecules using a QM model in the gas phase and Conceptual DFT^28^ (see “Methods” section). First, we use Δ*SCF* to compute the ionization potentials of the two molecules. For AFB_1_ the value is 7.3 eV and for AFG_2_ it is 7.5 eV. Thus, the two molecules appear equally easy to oxidize, from an energetics perspective. We further used the results of Δ*SCF* calculations to generate isosurfaces of the FuF which highlight the sites amenable to a hypothetical one-electron oxidation (Fig. 2). One significant difference between the two isosurfaces is the presence of an oxidation site on the furan ring of AFB_1_, which differs from AFG_2_ in that it has a double bond. This suggests that there is a different reactive site of AFB_1_ which is far from the lactone ring.

In the QM model, we see that the lactone ring does not spontaneously open after the toxin oxidation without an ulterior environmental stimulation, such as a hydrogen radical. A multi-step process is required to perform ring opening, which is reflected in the FuF, where we see that the electron is unlikely to be taken directly from the lactone ring (particularly in the case of AFB_1_). If such environmental stimulation is localized in the immediate proximity of the lactone ring, the structural rearrangement spontaneously ends in ring-opening (Supplementary Fig. S4). AFG_2_ exhibits a lower free-energy conformation post-ring breakdown (− 1.71 eV compared to the oxidized state), compared to AFB_1_ (− 1.34 eV compared to the oxidized state), suggesting a higher tendency towards this transition. However, as noted above, degradation affinity alone cannot describe the difference in dynamics between the two toxins.

**Figure 3:**
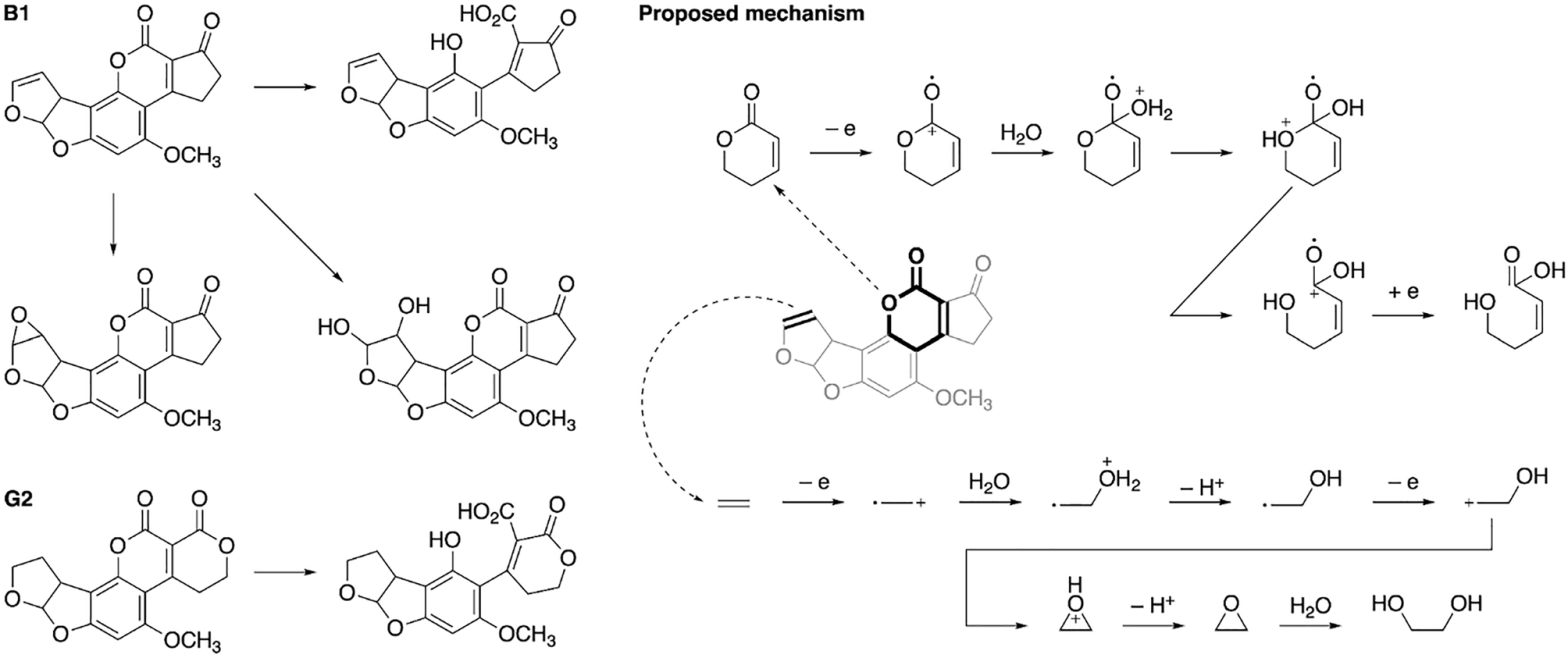
The main reaction products of AFB_1_ and AFG_2_ break-down, as identified by LC–MS and hypothetical reaction pathways. The main proposed mechanisms are the lactone ring opening and epoxide formation.

LC–MS analysis was performed to investigate the degradation products (Supplementary Figs. S2, S3). For both AFB_1_ and AFG_2_ a product with the ring open was found which matches the observation of reduced fluorescence (Fig. 3, including proposed mechanism). For AFB_1_, other oxidation products include the well-known epoxy- and dihydroxylated forms on the terminal furan ring (Supplementary Figs. S2, S3). An epoxy form of AFG_2_ was not found, which is consistent with our QM modeling which suggested that the furan ring of AFB_1_ is more reactive. This epoxy form would still fluoresce, and hence may represent an important side reaction which does not result in successful detoxification. The mechanism we propose is coherent with ammoniation of AFB_1_ to produce AFD_1_ through cleavage of the lactone ring, discussed in previous works^25,29,30^.

### Large scale modeling identifies building blocks of laccase-aflatoxin binding

**Figure 4:**
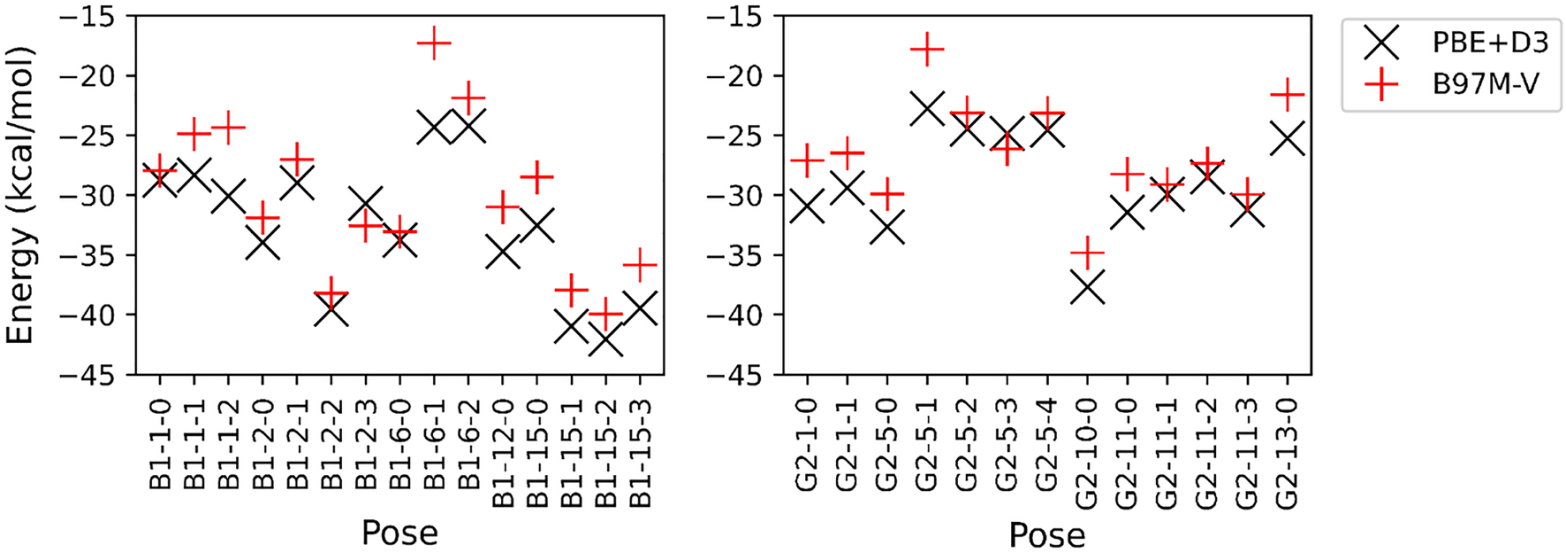
Interaction energies of different poses of AFB_1_ and AFG_2_ extracted from an MD simulation using a cluster model show no major differences between the congeners. Energies are shown for two different QM models (PBE + D3 and B97M-V).

Our experimental evidence has shown that TV laccase has insufficient affinity for both aflatoxins AFB_1_ and AFG_2_. To understand the binding observed, we utilized Docking and Molecular Dynamics (MD) to generate a diverse set of poses, which we can then score using DFT calculations (Fig. 4). Overall, we see little difference in the binding energies between AFB_1_ and AFG_2_, again supporting that energetics alone cannot explain the discrepancy in the dynamics between the two toxins. For AFG_2_, one pose (G-10-0) emerges as significantly lower in energy (Supplementary Fig. S5). We observe that AFG_2_ stays in this pose for the duration of that trajectory.

For AFB_1_, there are two competing low energy poses (B1-2-2 and B1-15-2), with the latter staying in a strong binding position for a longer duration. We note that while AFB_1_ may be oxidized in pose B1-2-2, it is unlikely that the ring is opened, because the reactive site of the lactone ring is buried deep in the pocket (Supplementary Fig. S5). From a purely geometric perspective, we also see that the furan ring of AFG_2_ is deep inside the pocket, whereas this site is exposed to the solvent for the low energy poses of AFB_1_. We found one additional pose of AFB_1_ (B1-1-0) which has the furan ring inside the pocket, though it has a weaker interaction energy than other AFB_1_ poses.

**Figure 5:**
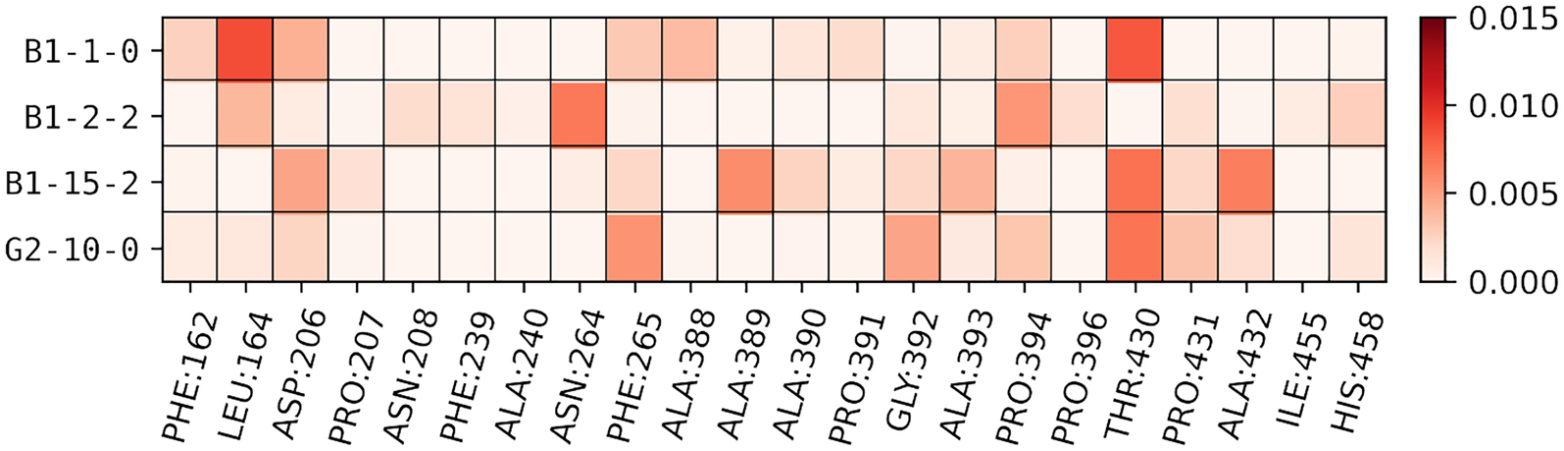
Heat map of interactions between laccase residues and low energy toxin poses as measured by FBO highlights residues of interest. A higher FBO indicates a stronger interaction.

In Fig. 5, we show a heat map of amino acid-toxin interactions as defined by the FBO measure. Visualizations of these interactions are available in Supplementary Fig. S6. For the sole low energy AFG_2_ pose, a strong interaction based on a hydrogen bond between Thr430 and the doubly bonded oxygen of the lactone ring is formed. Previous studies have brought attention to the role of Asn206 and His458 (the ligand of the type 1 blue copper) in charge transfer in laccase, with His458 participating in charge transfer and Asn206 playing a role in substrate recognition; it has been suggested that a distance of less than 5 Å is required for efficient degradation^31,32^. For G2-10-0, the oxygen of the furan ring is within 2.5 Å of His458 (and the toxin is 2.8 Å from Asp206), and is held there by an interaction with the neighboring Gly392. For the B1-1-0 pose of AFB_1_ (the pose most similar to G2-10-0), a similar interaction with Thr430 exists and is supplemented by an interaction with Leu164. In this pose, the oxygen of the furan ring is much further from His458 (4.4 Å), though the toxin is a similar distance to Asp206 (2.5 Å).

For B1-2-2, we note that the lactone ring is buried deep in the pocket, with the distance between the doubly bonded oxygen of the five-member ring and His458 being 2.4 Å. The primary interaction is between Asn264 and the furan rings on the opposite side. B1-2-2 also interacts with Pro394 which is close to His458, and is within 2.9 Å for Asp206. With B1-15-2, Thr430 is again an important interaction, as well as Ala389 and Ala432. B1-15-2 is close to Asp206 (2.4 Å), but far from His458; the methyl group is within 5.4 Å, and the nearest oxygen (of a furan group) is 6.6 Å away (the distances are similar for the other snapshots from the B1-15 trajectory). While the interaction with the enzyme is strong, this pose is likely poor for oxidation. We note that the only significant aromatic interaction detected in these low energy poses by our analysis involves Phe265. This residue interacts with the furan ring of B1-1-0 and G2-10-0, and not their aromatic groups. This suggests that there is ample opportunity for optimizing interactions of TV laccase with both AFB_1_ and AFG_2_ by exploiting π–π stacking.

## Discussion

Aflatoxin contamination is a major concern among food-safety issues, and laccase-mediated detoxification is viewed as a promising “green” bioremediation approach^12^. The evolution of this lignin-degrading enzyme has led to an active site with the highest redox potential among multi-copper oxidases^9^. Such uncommon oxidative potential is a necessary asset for breaking down the aromatic moieties of aflatoxins. However, as our data highlight, laccase lacks high affinity towards AFB_1_, and is therefore naturally far from optimized to carry out this reaction. For bioremediation to have a realistic chance at being consistently implemented, it has to be seamlessly incorporated during the current food production process. With this in mind, the best context for laccase-mediated aflatoxin bioremediation is during the conventional water-mediated washing step in the production process of food commodities. For that, AFB_1_ detoxification needs to be achieved in no longer than 3 h, in a slightly acidic (pH of 6.5), aerobic, liquid environment at room temperature. Our data indicate that, at pH 6.5, even the detoxification of AFG_2_ by TV laccase takes more than 48 h, a far cry from what would be practically required. Therefore, laccase as a realistic aflatoxin bioremediator requires substantial optimization.

Using a combination of experimental and multi-scale theoretical modeling, we find that high detail substrate-specific tuning is mandatory for application on aflatoxins. Such tuning will need to follow a different approach than even the best efforts at molecular docking (e.g. Refs.^33–35^). While it has been shown that with a sufficient set of descriptors from docking and MD simulations, along with QM modeling of the gas phase substrate^32^, it is possible to predict laccase affinity on a wide class of systems, the dramatic differences in dynamics between the two similar structures studied in this work show the limitation of a broader characterization. To this end, we have used a large-scale QM approach to identify the relevant amino acids involved in binding the low energy positions of the two toxins. Our results suggest that efficiency may be improved by optimizing laccase for stronger binding to aflatoxin, but care must be taken about the specific binding orientation that is being optimized. We suggest that tuning experiments begin with work on AFG_2_, as while AFB_1_ is a target of greater importance, the simpler dynamics of AFG_2_ will provide a clearer signal of a successful optimization workflow.

More research is still needed to fully elucidate the limitations of laccase for the bioremediation of aflatoxins. Laccase is particularly difficult to model, using either classical MD or DFT, due to the presence of a transition metal. More sophisticated advanced sampling may also reveal a wider set of possible binding poses. A full QM/MM study of the detoxification process in the laccase pocket should be performed in the future. These simulation studies may be combined with further experimental work, such as Small Angle X-ray Scattering (SAXS) and Small Angle Neutron Scattering (SANS) which have the ability to probe the structure of laccase at finite temperature^10,36^. Despite these limitations, our study has found strong evidence of a need for careful optimization of the laccase pocket, and specific directions to improve efficacy. For such a rational design project, the residues identified in this study, along with the general insights into the degradation process presented here, will make a good starting point.

## Methods

### Modeling the detoxification of aflatoxins by laccase

We assume that laccase detoxifies aflatoxins following the Michaelis–Menten equation:

2 $$\frac{d\left[T\right]}{dt}=-{k}_{L}\left[L\right]\frac{\left[T\right]}{{K}_{m}+\left[T\right]},$$

in which $\left[T\right]$ is the toxin concentration (in $\frac{\mu \mathrm{g}}{\mathrm{mL}}$), $\left[L\right]$ is the laccase concentration (in $\mathrm{U}/\mathrm{mL}$), ${K}_{m}$ is the Michaelis–Menten constant (in $\mu \mathrm{g}/\mathrm{mL}$), and ${k}_{L}$ is the degradation rate by laccase from the enzyme-toxin associated state (in $\mu \mathrm{g}/(\mathrm{mL}\cdot \mathrm{h})$). In the limit that the toxin concentration is much lower than the Michaelis–Menten constant (${K}_{m}\gg \left[T\right]$), the equation will be simplified to

3 $$\frac{d\left[T\right]}{dt}\approx -\frac{{k}_{L}}{{K}_{m}}\left[L\right]\left[T\right].$$

We define the detoxification efficiency as $\eta \stackrel{\scriptscriptstyle\mathrm{def}}{=}\frac{-1}{\left[L\right]\left[T\right]}\frac{\Delta \left[T\right]}{\Delta t}\approx \frac{{k}_{L}}{{K}_{m}}$ (in $\mathrm{mL}/(\mathrm{U}\cdot \mathrm{h}))$, which can be calculated from the experimental data. Here $\Delta \left[T\right]$ is the change in the toxin concentration in a small time-step $\Delta t$. Since we can measure $\left[T\right]$ experimentally over time, we can calculate $\eta$ as well as the local normalized detoxification rate $\rho =\frac{-1}{\left[L\right]}\frac{d\left[T\right]}{dt}$. When $\eta =\frac{\rho }{[T]}$ is constant, we can infer that ${K}_{m}\gg \left[T\right]$.

### Fluorescence-based assay of laccase-mediated detoxification of AFB_1_ and AFG_2_

Laccase from *Trametes versicolor* (Merck CAS80498) is dissolved in acetate buffer (pH 6.5) at a final concentration of 25 U/mL. Aflatoxin B_1_ and Aflatoxin G_2_ (Cayman Chemicals) are dissolved in LC–MS grade methanol (Merck) at 4 different concentrations: 3, 30, 50, 100 μg/mL. Buffer solutions of laccase and aflatoxins are incubated at 28 °C, over 96 h under the fast continuous shaking regime in a Synergy™ Mx Multi-Mode Microplate Reader (Biotek); each condition is performed in triplicate. Due to its natural fluorescence, aflatoxin concentration is fluorimetrically assayed (excitation at 380 nm and emission at 440 nm; gain 65 and 50 for AFB_1_ and AFG_2_, respectively); readouts are acquired every 10 min, totaling 577 by the end of the experiment. Controls assay laccase fluorescence in the buffer in the absence of aflatoxins, and AFB_1_ and AFG_2_ fluorescence in the absence of laccase. To convert the fluorescence readout to the corresponding toxin concentration, we employ a calibration curve based on measurements of a set of known toxin concentrations (Supplementary Fig. S1).

The reaction kinetics are simulated in Matlab. The method of least squares (lsqnonlin in Matlab) is employed to fit the Michaelis–Menten kinetics to the experimental data. Detoxification efficiency is estimated by using the data from the first 100 min for each experiment, and finding the best linear fit (no intercept) as a function of the initial toxin concentration.

### Identification of degradation products of laccase activity on aflatoxins via LC/MS

25 U/mL laccase from Trametes versicolor (Sigma-Aldrich CAS80498) was added to 10 μg/mL of toxin, AFB_1_ or AFG_2_ (both from Cayman Chemicals) separately, in acetate buffer (100 mM, pH 6.5) and left at 28 °C for 24 h. Degradation products were assayed under the following conditions: Column: Kinetex 2.6 μm EVO C18; 100 × 2.1 mm; Mobile phase A: Water 5 mM Ammonium Acetate, 0.5% Acetic Acid; Mobile phase B: Methanol 5 mM Ammonium Acetate, 0.5% Acetic Acid; Flow rate: 350 μL/min; UV Wavelength: 354, 360 nm.

The following gradient method was used in all runs. The eluent from the column was directed into the electrospray source of an Agilent 6220 TOF mass spectrometer operated in positive ionization mode. Data was converted into the mzML file format and analyzed using the MZMine software. Supplementary Figs. S2 and S3 show the resulting traces for AFB_1_ and AFG_2_, respectively. AFB_1_ and AFG_2_ detoxification byproducts are shown in the supplements.

### Density functional theory (DFT) calculations

QM calculations are performed within the framework of Kohn–Sham Density Functional Theory (KS-DFT)^37^, employing the Perdew–Burke–Ernzerhof (PBE)^38^ exchange and correlation level of theory. The numerical results are extracted with the BigDFT code^39^, which uses Daubechies wavelets to express the KS orbitals. Hartwigsen–Goedecker–Hutter (HGH) pseudopotentials^40^ are used to remove the orbitals in the core electrons. The use of a wavelet basis sets enables systemic control of the resulting accuracy. Isolated boundary conditions are explicitly included in the calculations, without supercell aliasing effects, using the Poisson Solver of the code^41^. A wavelet grid spacing of 0.37 atomic units is employed for the calculations presented in this work. The code performs charged Δ*SCF* calculations, and the Fukui functions (FuF) are defined as the difference between the neutral ground state electronic density and the corresponding quantity in the vertical cationic state.

### Docking

The initial 3D crystallographic structure of the *Trametes versicolor* beta isoform, based on a structure from the Protein DataBank (accession code 1KYA)^42^, is taken from previous work. The protein model is cleaned up using the pdb4amber script, part of the Amber 2020 software suite. Once the structure passes inspection by Amber’s LEaP program, it is protonated using the H++ webserver (version 3.2)^43–45^ for a target pH of 6.5 to reflect conditions feasible in application in the context of the food industry production process. Because H++ does not account for metals, the resultant protonated structure is manually cleaned to flip histidine residues in order to maintain proper ligation of the embedded copper atoms. No explicit solvent molecules are included in the final structures. Ligand 3D structures are generated from ChemDraw 19.1 and optimized with Gaussian16^46^ in gas phase using HF/6-31G*. The resultant geometries are imported in Hermes, an application component of the CSD-Discovery Suite 2020 which interfaces with GOLD^47^. The bonds are repaired using Hermes’ structure clean up to ensure readability by GOLD.

The protonated and adjusted protein structures are imported into the GOLD 2020.2.0 docking setup wizard. The pocket is defined using an atom from a residue lining the cavity, and GOLD’s pocket finding algorithm is used to determine the pocket. Care is taken to ensure all residues and the copper atoms are appropriately recognized. All ligand and receptor flexibility options are enabled in addition to diverse solutions with a cluster size of 5 and a root-mean-square deviation (RMSD) of 2 Å. The genetic algorithm is set to the maximum search efficiency with automatic settings for the algorithm itself. Poses are scored and rescored with CHEMPLP and GoldScore, respectively. From the top 15 docked poses of each toxin, we visually inspect and extract five poses that represent unique binding orientations (see Supplementary Table S1 for docking scores).

### Molecular dynamics (MD)

Molecular Dynamics simulations start from each of the five extracted docked poses for each of the toxins. Simulations are performed using the OpenMM framework^48^ with the FF14SB forcefield^49^ for laccase, and the SMIRKS Native Open Force Field^50^ for each toxin. We use a temperature of 300 K, plus the Variable Langevin Integrator with the default convergence and friction coefficients for 20,000,000 steps (approximately 25 ns). We constrain the distances between copper atoms and neighboring histidine (nitrogen) and cysteine (sulfur) residues amongst the type 2 and type 3 coppers, based on a set of optimized geometries (see the following section). We discard the early parts of each simulation using the marginal standard error rule heuristic with a batch size of 200^51,52^, as well as the snapshots where aflatoxin leaves the pocket (B1-6-3, B1-6-4, G2-1-2, G2-13-1, G2-13-2, G2-13-3). Using the embedding environments defined in the following section as a guide, we extract snapshots from the MD simulations. The residues in the environment are aligned, and a new snapshot is extracted whenever the root mean square deviation between a given snapshot and all extracted snapshots is greater than 4.0 bohr.

We caution that such an MD protocol is by itself insufficient for free energy calculations. The copper atoms and the cysteine radical of laccase are challenging to model, and require a specially parameterized forcefield, or QM/MM dynamics (see Refs.^53,54^ for laccase). Longer trajectory times, or advanced sampling techniques, are also required for a full sampling of the configuration space. We emphasize that our MD simulations is intended to generate a diverse set of plausible aflatoxin poses to post-process with QM calculations.

### Embedding environment generation

We previously described a Complexity Reduction framework which uses the electronic density computed by QM calculations to represent a full system from calculations of only a subset of the system^55,56^. The key step in this analysis is computation of the Fragment Bond Order (FBO), which is a generalization of atomic bond order to interactions between two arbitrary sets of atoms. The FBO, in this case, is used to assign an interaction strength (unit-less) to amino acid-ligand pairs. The FBO can then be used to generate an embedding environment, defined as the minimal set of fragments such that the sum of the bond orders of all excluded fragments is below a threshold, set at 0.01 for this study. The ability to break down protein–ligand interactions into a per amino acid contribution is a strong asset of large scale QM calculations^57^.

To compute the FBO, we perform DFT calculations on the entire protein-toxin complex (nearly 7400 atoms) using the linear scaling mode of BigDFT^58–60^, with implicit solvent^61^. We perform calculations on both the docked structures and the snapshots from the MD simulations. For the docked structures, we perform initial optimization to remove steric clashes challenging for DFT calculations. First, the positions of the hydrogen atoms are optimized using the FF14SB force-field. Second, each residue of the protein is optimized using the GFN2 tight binding method^62^, with implicit solvent. Each residue is embedded in a hydrogen capped fixed environment of residues defined as those within 4.5 Å, and then optimized. For the copper atoms, a similar embedding strategy was used, with the neighboring histidines also allowed to move. To optimize the trinuclear cluster, we construct a system containing all three coppers and residues within 4.5 Å of any of the three coppers, with all histidines and copper atoms allowed to move.

From calculations of the docked structures, we used the FBO to identify a set of residues interacting strongly with the toxin in at least one pose: Phe162, Pro163, Leu164, Asp206, Asn264, Phe265, Phe332, Phe337, Pro391, Gly392, Ala393, Pro394, Ile455, His458. These amino acids are tracked when extracting snapshots from the MD simulation. We utilize this same FBO approach to construct cluster models from extracted MD snapshots. We identify residues, strongly interacting with either the toxin or the copper atom of the active site, to be added to the model. We further include any connecting amino acids and terminate the clusters with amide caps. The cluster system includes residues: Lys157, Pro160, Ala161, Phe162, Pro163, Leu164, Asp206, Pro207, Asn208, Asn264, Phe265, Thr335, Asn336, Phe337, Ala388, Ala389, Ala390, Pro391, Gly392, Ala393, Pro394, His395, Thr430, Pro431, Ala432, Cys453, His454, Ile455, Asp456, Phe457, His458, Leu459, Glu460, Ala461.

### Interaction energies calculations

Interaction energies are computed in the above defined embedding environment with a three point approach using the PBE functional with dispersion corrections^63^, and implicit solvent. A smaller cluster system enables us to use the cubic scaling mode of BigDFT which can converge to a higher accuracy than the linear scaling mode. We further refine the energies using the B97M-V functional in conjunction with the def2-mSVP basis set^64^, as implemented in Orca^65^. The B97M-V method has recently been shown to yield accurate binding energies between an enzyme and its substrate for a wide range of systems^66^. The geometric counter poise (gCP) correction^67^, parameterized for the def2-mSVP basis set, is applied to provide an accuracy that is comparable to that at the complete-basis-set limit. The B97M-V/def2-mSVP calculations were carried out with aqueous solvation effects included by using the CPCM model^68^.

## Supplementary Information

Supplementary Information.

## Data Availability

The source codes for the reaction kinetics analysis are available on GitHub (https://github.com/bmomeni/laccase-aflatoxins-reaction-kinetics). The BigDFT code for performing QM calculations and the PyBigDFT code for computing the FBO are available on the BigDFT website (https://www.bigdft.org/).

## References

[1] Bennett JW, Klich M (2003). Mycotoxins. Clin. Microbiol. Rev..

[2] Chu FS, Caballero B, Trugo LC, Finglas PM (2003). Toxicology. Encyclopedia of Food Sciences and Nutrition.

[3] Klingelhöfer D (2018). Aflatoxin—Publication analysis of a global health threat. Food Control.

[4] Lyagin I, Efremenko E (2019). Enzymes for detoxification of various mycotoxins: Origins and mechanisms of catalytic action. Molecules.

[5] Wu Q (2009). Biological degradation of aflatoxins. Drug Metab. Rev..

[6] Alberts JFF, Gelderblom WCACA, Botha A, van Zyl WHH (2009). Degradation of aflatoxin B1 by fungal laccase enzymes. Int. J. Food Microbiol..

[7] Dellafiora L, Galaverna G, Reverberi M, Dall’Asta C (2017). Degradation of aflatoxins by means of laccases from trametes versicolor: An in silico insight. Toxins (Basel).

[8] Scarpari M (2014). Aflatoxin control in maize by Trametes versicolor. Toxins (Basel)..

[9] Mate DM, Alcalde M (2017). Laccase: A multi-purpose biocatalyst at the forefront of biotechnology. Microb. Biotechnol..

[10] Zaccaria M (2020). Designing a bioremediator: Mechanistic models guide cellular and molecular specialization. Curr. Opin. Biotechnol..

[11] Solomon EI, Sundaram UM, Machonkin TE (1996). Multicopper oxidases and oxygenases. Chem. Rev..

[12] Alcalde M, Polain AJ, MacCabe AP (2007). Laccases: Biological functions, molecular structure and industrial applications. Industrial Enzymes: Structure, Function and Applications.

[13] Wang X (2019). Degradation of aflatoxin B1 and zearalenone by bacterial and fungal laccases in presence of structurally defined chemicals and complex natural mediators. Toxins.

[14] Guo Y (2020). CotA laccase, a novel aflatoxin oxidase from *Bacillus licheniformis*, transforms aflatoxin B1 to aflatoxin Q1 and epi-aflatoxin Q1. Food Chem..

[15] Kumar V (2021). Recent technological advances in mechanism, toxicity, and food perspectives of enzyme-mediated aflatoxin degradation. Crit. Rev. Food Sci. Nutr..

[16] Liu Y (2021). Degradation of aflatoxin B1 by a recombinant laccase from Trametes sp. C30 expressed in *Saccharomyces cerevisiae*: A mechanism assessment study in vitro and in vivo. Food Res. Int..

[17] Okwara PC, Afolabi IS, Ahuekwe EF (2021). Application of laccase in aflatoxin B1 degradation: A review. IOP Conf. Ser. Mater. Sci. Eng..

[18] Festa G, Autore F, Fraternali F, Giardina P, Sannia G (2008). Development of new laccases by directed evolution: Functional and computational analyses. Proteins Struct. Funct. Bioinform..

[19] Mate DM, Alcalde M (2015). Laccase engineering: From rational design to directed evolution. Biotechnol. Adv..

[20] Mateljak I (2019). Increasing redox potential, redox mediator activity, and stability in a fungal laccase by computer-guided mutagenesis and directed evolution. ACS Catal..

[21] Liu Y (2020). Molecular docking studies and in vitro degradation of four aflatoxins (AFB1, AFB2, AFG1, and AFG2) by a recombinant laccase from *Saccharomyces cerevisiae*. J. Food Sci..

[22] Zhou Z, Li R, Ng TB, Huang F, Ye X (2022). Considerations regarding affinity determinants for aflatoxin B1 in binding cavity of fungal laccase based on in silico mutational and in vitro verification studies. Ecotoxicol. Environ. Saf..

[23] Bourbonnais R, Paice MG, Reid ID, Lanthier P, Yaguchi M (1995). Lignin oxidation by laccase isozymes from trametes versicolor and role of the mediator 2,2’-azinobis(3-ethylbenzthiazoline-6-sulfonate) in kraft lignin depolymerization. Appl. Environ. Microbiol..

[24] Hakulinen N, Rouvinen J (2015). Three-dimensional structures of laccases. Cell. Mol. Life Sci..

[25] Lee LS, Dunn JJ, DeLucca AJ, Ciegler A (1981). Role of lactone ring of aflatoxin B1 in toxicity and mutagenicity. Experientia.

[26] Nelson DL, Cox MM (2017). Lehninger Principles of Biochemistry.

[27] Zeinvand-Lorestani H (2015). Comparative study of in vitro prooxidative properties and genotoxicity induced by aflatoxin B1 and its laccase-mediated detoxification products. Chemosphere.

[28] Geerlings P, De Proft F, Langenaeker W (2003). Conceptual density functional theory. Chem. Rev..

[29] Lee LS, Cucullu AF (1978). Conversion of aflatoxin B1 to aflatoxin D1 in ammoniated peanut and cottonseed meals. J. Agric. Food Chem..

[30] Motomura M, Toyomasu T, Mizuno K, Shinozawa T (2003). Purification and characterization of an aflatoxin degradation enzyme from *Pleurotus ostreatus*. Microbiol. Res..

[31] Christensen NJ, Kepp KP (2014). Setting the stage for electron transfer: Molecular basis of ABTS-binding to four laccases from Trametes versicolor at variable pH and protein oxidation state. J. Mol. Catal. B Enzym..

[32] Mehra R, Muschiol J, Meyer AS, Kepp KP (2018). A structural-chemical explanation of fungal laccase activity. Sci. Rep..

[33] Awasthi M, Jasiwal N, Singh S, Pandey VP, Dwivedi UN (2014). Molecular docking and dynamics simulation analyses unraveling the differential enzymatic catalysis by plant and fungal laccases with respect to lignin biosynthesis and degradation. J. Biomol. Struct. Dyn..

[34] Martínez-Sotres C, Rutiaga-Quiñones JG, Herrera-Bucio R, Gallo M, López-Albarrán P (2015). Molecular docking insights into the inhibition of laccase activity by medicarpin. Wood Sci. Technol..

[35] Kadam SK, Tamboli AS, Sambhare SB, Jeon BH, Govindwar SP (2018). Enzymatic analysis, structural study and molecular docking of laccase and catalase from *B. subtilis* SK1 after textile dye exposure. Ecol. Inform..

[36] Zaccai NR, Coquelle N (2020). Opportunities and challenges in neutron crystallography. EPJ Web Conf..

[37] Kohn W, Sham LJ (1965). Self-consistent equations including exchange and correlation effects. Phys. Rev..

[38] Perdew JP, Burke K, Ernzerhof M (1996). Generalized gradient approximation made simple. Phys. Rev. Lett..

[39] Ratcliff LE (2020). Flexibilities of wavelets as a computational basis set for large-scale electronic structure calculations. J. Chem. Phys..

[40] Willand A (2013). Norm-conserving pseudopotentials with chemical accuracy compared to all-electron calculations. J. Chem. Phys..

[41] Cerioni A, Genovese L, Mirone A, Sole VA (2012). Efficient and accurate solver of the three-dimensional screened and unscreened Poissons equation with generic boundary conditions. J. Chem. Phys..

[42] Bertrand T (2002). Crystal structure of a four-copper laccase complexed with an arylamine: Insights into substrate recognition and correlation with kinetics. Biochemistry.

[43] Anandakrishnan R, Aguilar B, Onufriev AV (2012). H++ 3.0: Automating pK prediction and the preparation of biomolecular structures for atomistic molecular modeling and simulations. Nucleic Acids Res..

[44] Myers J, Grothaus G, Narayanan S, Onufriev A (2006). A simple clustering algorithm can be accurate enough for use in calculations of pKs in macromolecules. Proteins Struct. Funct. Genet..

[45] Gordon JC (2005). H++: A server for estimating pKas and adding missing hydrogens to macromolecules. Nucleic Acids Res..

[46] Frisch, M. J. *et al. Gaussian 16 Revision A.03* (2016).

[47] Jones G, Willett P, Glen RC, Leach AR, Taylor R (1997). Development and validation of a genetic algorithm for flexible docking. J. Mol. Biol..

[48] Eastman P (2017). OpenMM 7: Rapid development of high performance algorithms for molecular dynamics. PLoS Comput. Biol..

[49] Maier JA (2015). ff14SB: Improving the accuracy of protein side chain and backbone parameters from ff99SB. J. Chem. Theory Comput..

[50] Mobley DL (2018). Escaping atom types in force fields using direct chemical perception. J. Chem. Theory Comput..

[51] White KP (2016). An effective truncation heuristic for bias reduction in simulation output. Simulation.

[52] Dawson W, Gygi F (2018). Equilibration and analysis of first-principles molecular dynamics simulations of water. J. Chem. Phys..

[53] Santiago G (2016). Computer-aided laccase engineering: Toward biological oxidation of arylamines. ACS Catal..

[54] Monza E (2015). Insights into laccase engineering from molecular simulations: Toward a binding-focused strategy. J. Phys. Chem. Lett..

[55] Mohr S, Masella M, Ratcliff LE, Genovese L (2017). Complexity reduction in large quantum systems: Fragment identification and population analysis via a local optimized minimal basis. J. Chem. Theory Comput..

[56] Dawson W, Mohr S, Ratcliff LE, Nakajima T, Genovese L (2020). Complexity reduction in density functional theory calculations of large systems: system partitioning and fragment embedding. J. Chem. Theory Comput..

[57] Dawson W (2021). Density functional theory calculations of large systems: Interplay between fragments, observables, and computational complexity. Wiley Interdiscip. Rev. Comput. Mol. Sci..

[58] Mohr S (2014). Daubechies wavelets for linear scaling density functional theory. J. Chem. Phys..

[59] Mohr S (2015). Accurate and efficient linear scaling DFT calculations with universal applicability. Phys. Chem. Chem. Phys..

[60] Mohr S (2017). Efficient computation of sparse matrix functions for large-scale electronic structure calculations: The CheSS library. J. Chem. Theory Comput..

[61] Fisicaro G, Genovese L, Andreussi O, Marzari N, Goedecker S (2016). A generalized Poisson and Poisson-Boltzmann solver for electrostatic environments. J. Chem. Phys..

[62] Bannwarth C, Ehlert S, Grimme S (2019). GFN2-xTB—An accurate and broadly parametrized self-consistent tight-binding quantum chemical method with multipole electrostatics and density-dependent dispersion contributions. J. Chem. Theory Comput..

[63] Grimme S (2006). Semiempirical GGA-type density functional constructed with a long-range dispersion correction. J. Comput. Chem..

[64] Grimme S, Brandenburg JG, Bannwarth C, Hansen A (2015). Consistent structures and interactions by density functional theory with small atomic orbital basis sets. J. Chem. Phys..

[65] Neese F (2022). Software update: The ORCA program system—Version 5.0. Wiley Interdiscip. Rev. Comput. Mol. Sci..

[66] Chan B, Dawson W, Nakajima T (2022). Searching for a reliable density functional for molecule-environment interactions, found B97M-V/def2-mTZVP. J. Phys. Chem. A.

[67] Kruse H, Grimme S (2012). A geometrical correction for the inter- and intra-molecular basis set superposition error in Hartree–Fock and density functional theory calculations for large systems. J. Chem. Phys..

[68] Barone V, Cossi M (1998). Quantum calculation of molecular energies and energy gradients in solution by a conductor solvent model. J. Phys. Chem. A.

