# Supplementary material for: Experimental–theoretical study of laccase as a detoxifier of aflatoxins
Source: Sci Rep. 2023 Jan 17;13:860. doi: 10.1038/s41598-023-27519-1 (PMC9845376; doi:10.1038/s41598-023-27519-1)
Supplement: Supplementary file 1 — Supplementary Information. [file 41598_2023_27519_MOESM1_ESM.pdf]

Supplementary Information

Experimental-Theoretical Study of Laccase as a Detoxifier of Aflatoxins

Marco Zaccaria+, William Dawson+, Darius Russel Kish, Massimo Reverberi, Maria Carmela Bonaccorsi di Patti, Marek Domin, Viviana Cristiglio, Bun Chan, Luca Dellafiora, Frank Gabel, Takahito Nakajima, Luigi Genovese, and Babak Momeni*

* Corresponding author: momeni@bc.edu

+ These authors contributed equally to this work

Calibration curves for fluorescence-based aflatoxin detoxification assay

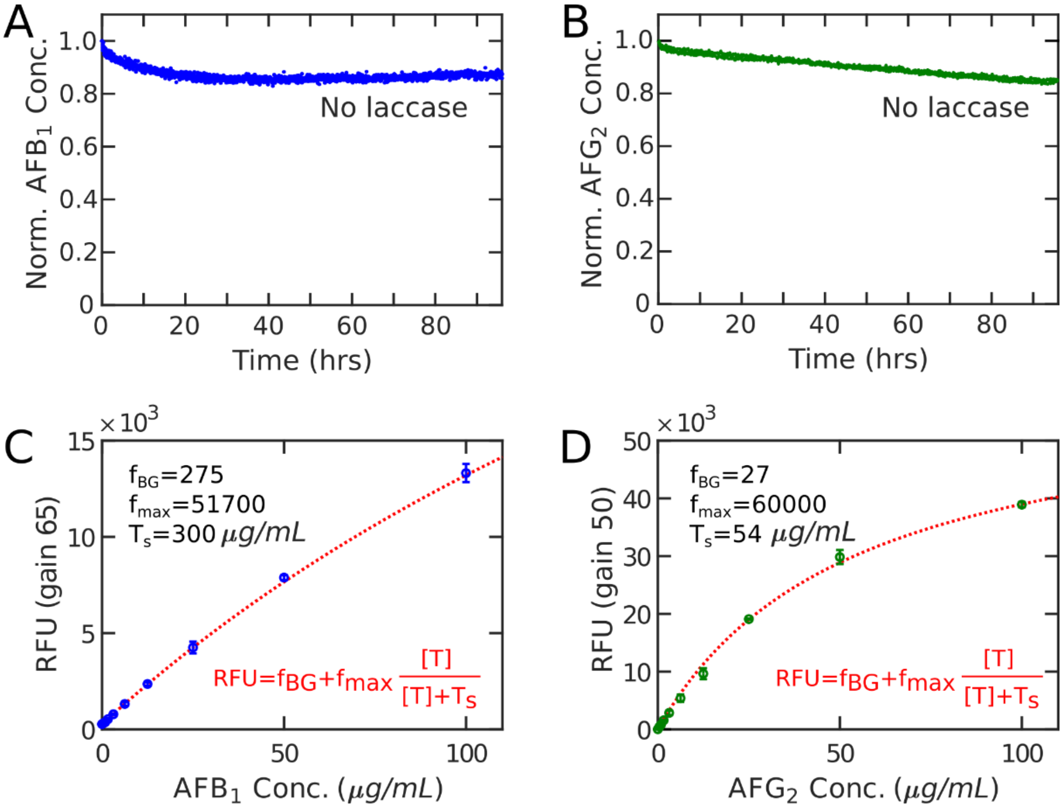

Supplementary Fig. S1. Calibration curves for fluorescence-assayed toxin concentration show reliable conversion between fluorescence and aflatoxin concentration. (A-B) In the absence of laccase, little loss of fluorescence is observed, suggesting that laccase is the main driver of aflatoxins detoxification in our setup. (C-D) [T] represents the toxin concentration; RFU represents Relative Fluorescence Units; T_s, f_{BG} and f_{max} are fitting parameters; for fitting the parameters, we used the method of least squares as implemented in Matlab.

Different starting concentrations of aflatoxin aflatoxin B_1 (AFB_1) and aflatoxin G_2 (AFG_2) (both from Cayman Chemicals) in acetate buffer (100 mM, pH 6.5) were employed to develop calibration curves to correlate measured fluorescence (ex. 380 nm; em. 440 nm) to toxin concentration. A SynergyTM Mx Multi-Mode Microplate Reader (BioTek) was used to perform the measurements.

In the absence of laccase, the fluorescence in control wells only slightly decreased (Fig. S1, A-B), suggesting little non-enzymatic degradation of aflatoxins. Read-outs in other cells were divided by this no-laccase controls to correct for the background decrease in fluorescence, presumably due to repeated exposure to the excitation light during the course of the assay.

Identification of degradation products of laccase activity on aflatoxins via LC/MS

50 U/mL laccase from Trametes versicolor (Sigma-Aldrich CAS80498) were added to 10 μg/mL of toxin, AFB_1 or AFG_2 (both from Cayman Chemicals) separately, in acetate buffer (100 mM, pH 6.5) and left at 28°C for 24 hrs. Degradation products were assayed under the following conditions:
Column: Kinetex 2.6 μm EVO C18; 100 x 2.1 mm.
Mobile phase A: Water 5 mM Ammonium Acetate, 0.5% Acetic Acid.
Mobile phase B: Methanol 5 mM Ammonium Acetate, 0.5% Acetic Acid.
Flow rate: 350 μL/min.
UV Wavelength: 354, 360 nm

The following gradient method was used in all runs.

Time (min)	%A	%B
Initial	90	10
3	90	10
10	30	70
10.1	10	90
12	10	90
12.1	90	10
15	90	10

The eluent from the column was directed into the electrospray source of an Agilent 6220 TOF mass spectrometer operated in positive ionization mode. Data was converted into the mzML file format and analyzed using the MZMine software.

Supplementary Figs. S2 and S3 show the resulting traces for AFB_1 and AFG_2, respectively.

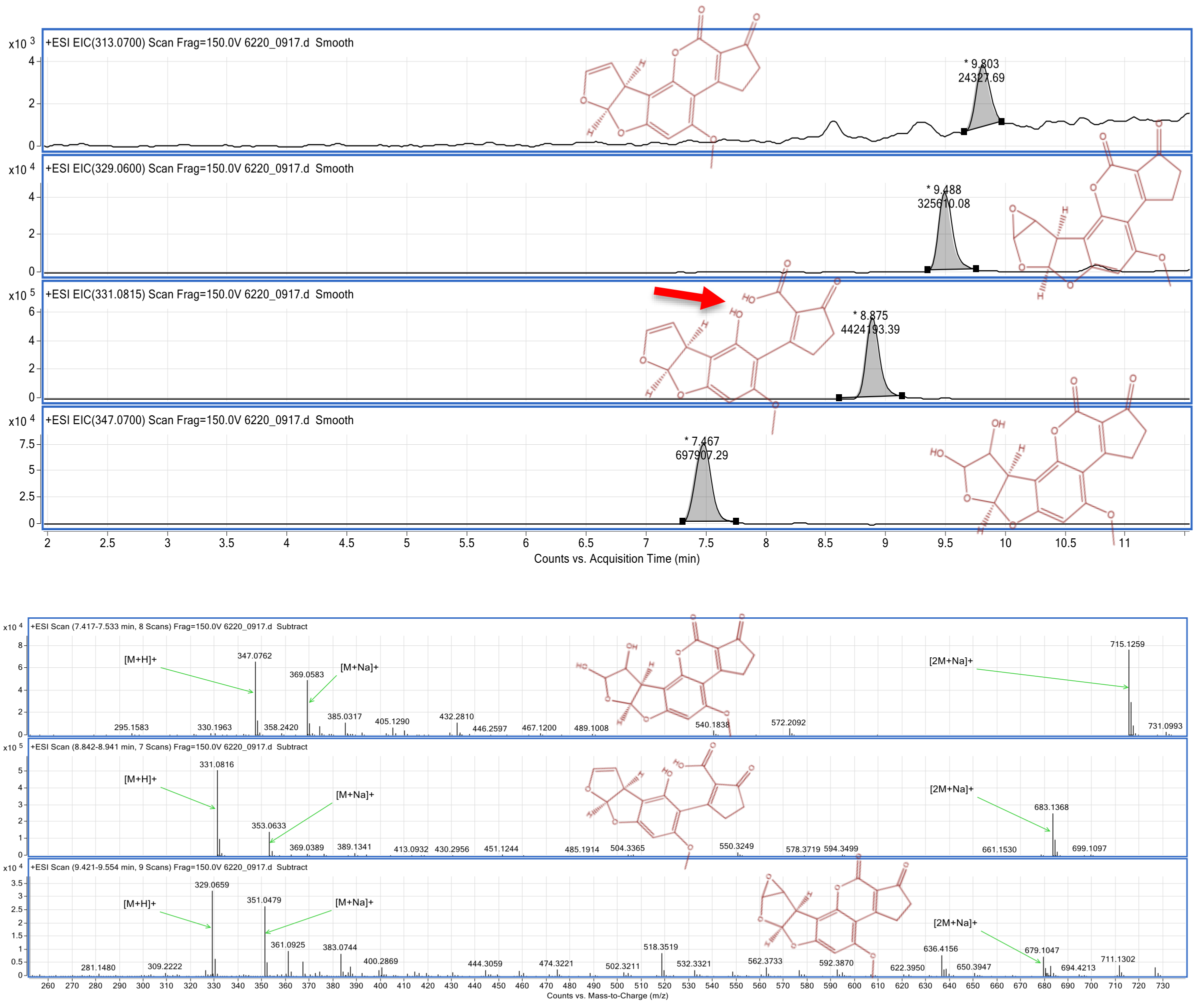

Meas. mass u	Abund. %	Diff. ppm	Unsat.	Compositions
347.076202	0.00	-1.41	10.5	C17 H15 O8

Meas. mass u	Abund. %	Diff. ppm	Unsat.	Compositions
331.081604	0.00	-0.52	10.5	C17 H15 O7

Meas. mass u	Abund. %	Diff. ppm	Unsat.	Compositions
329.065887	0.00	-0.75	11.5	C17 H13 O7

Supplementary Fig. S2. Mass spectroscopy reveals byproducts of AFB$_1$ detoxification. The fully detoxified AFB$_1$ molecule is the one with an open lactone ring, highlighted by the red arrow.

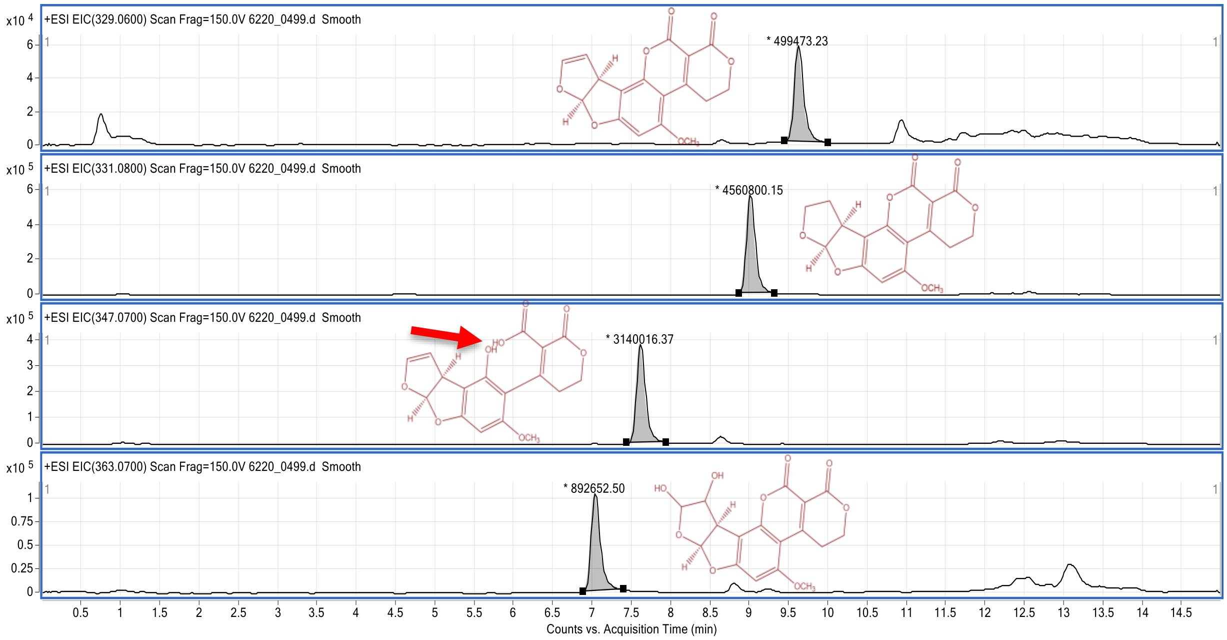

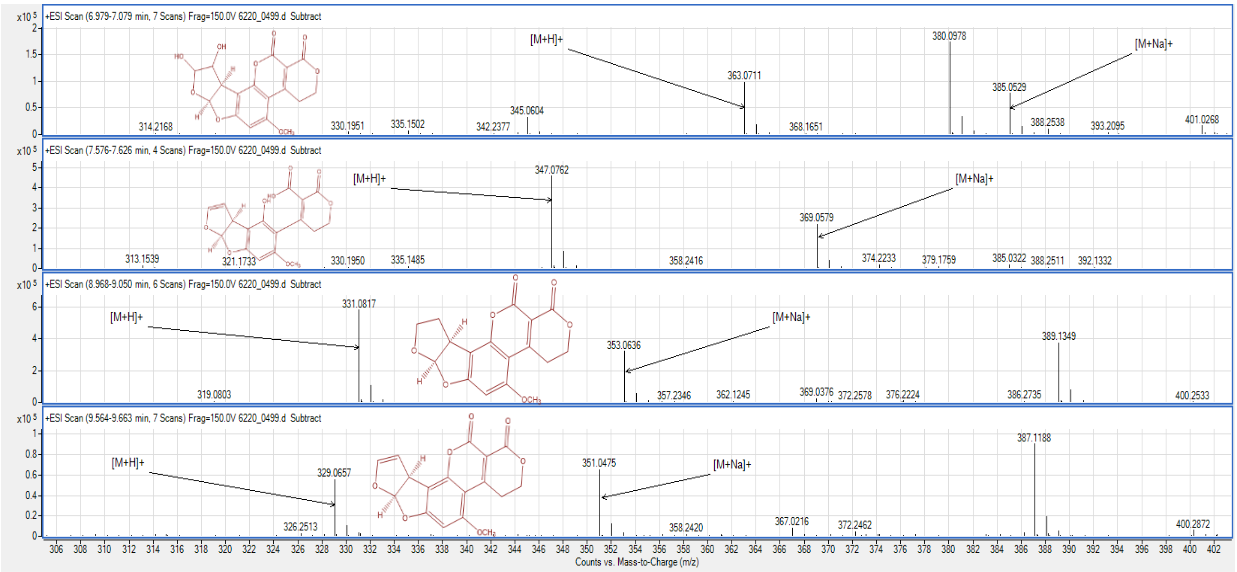

Meas. mass u	Abund. %	Diff. ppm	Unsat.	Compositions
331.081696	0.00	-0.24	10.5	C17 H15 O7

Meas. mass u	Abund. %	Diff. ppm	Unsat.	Compositions
347.075592	0.00	-3.17	10.5	C17 H15 O8

Meas. mass u	Abund. %	Diff. ppm	Unsat.	Compositions
363.071289	0.00	-0.87	10.5	C17 H15 O9

Meas. mass u	Abund. %	Diff. ppm	Unsat.	Compositions
329.065491	0.00	-1.96	11.5	C17 H13 O7

Supplementary Fig. S3. Mass spectroscopy reveals byproducts of AFG$_2$ detoxification. The fully detoxified AFG$_2$ molecule is the one with an open lactone ring, highlighted by the red arrow.

Ring Opening

An image of ring opening of the two toxins is shown in Supplementary Fig. S4.

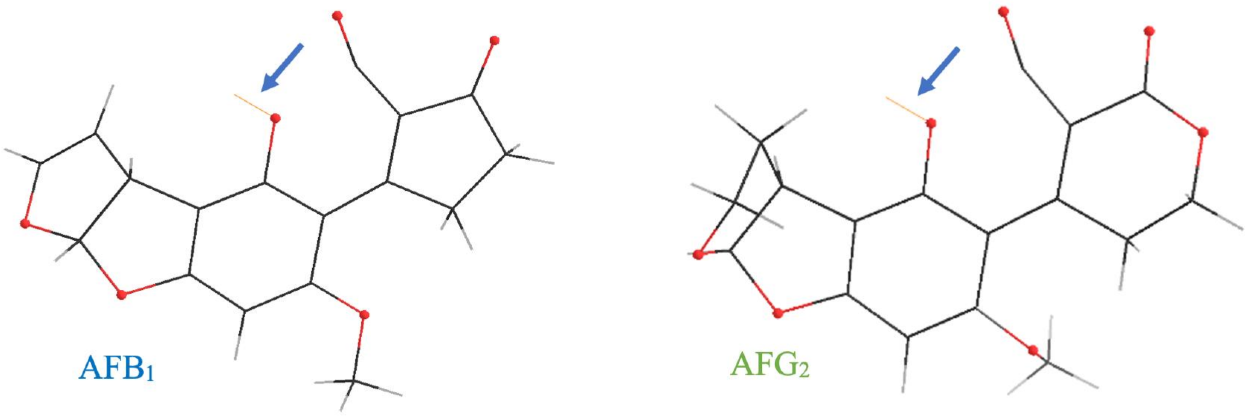

Supplementary Fig. S4. Conformations of AFB1 and AFG2 during the simulated lactone-ring opening process indicate the position of environmental stimulation. We present a snapshot of the geometry optimization procedure of the oxidized aflatoxin and H·system, showing how this would lead to a ring opening. The attacking hydrogen is shown by the yellow bond.

Images of Active Sites

Images of the active sites of low energy conformers were generated with PyMOL and are shown in Supplementary Fig. S5. We further show those conformations with only the strongly interacting residues in Supplementary Fig. S6.

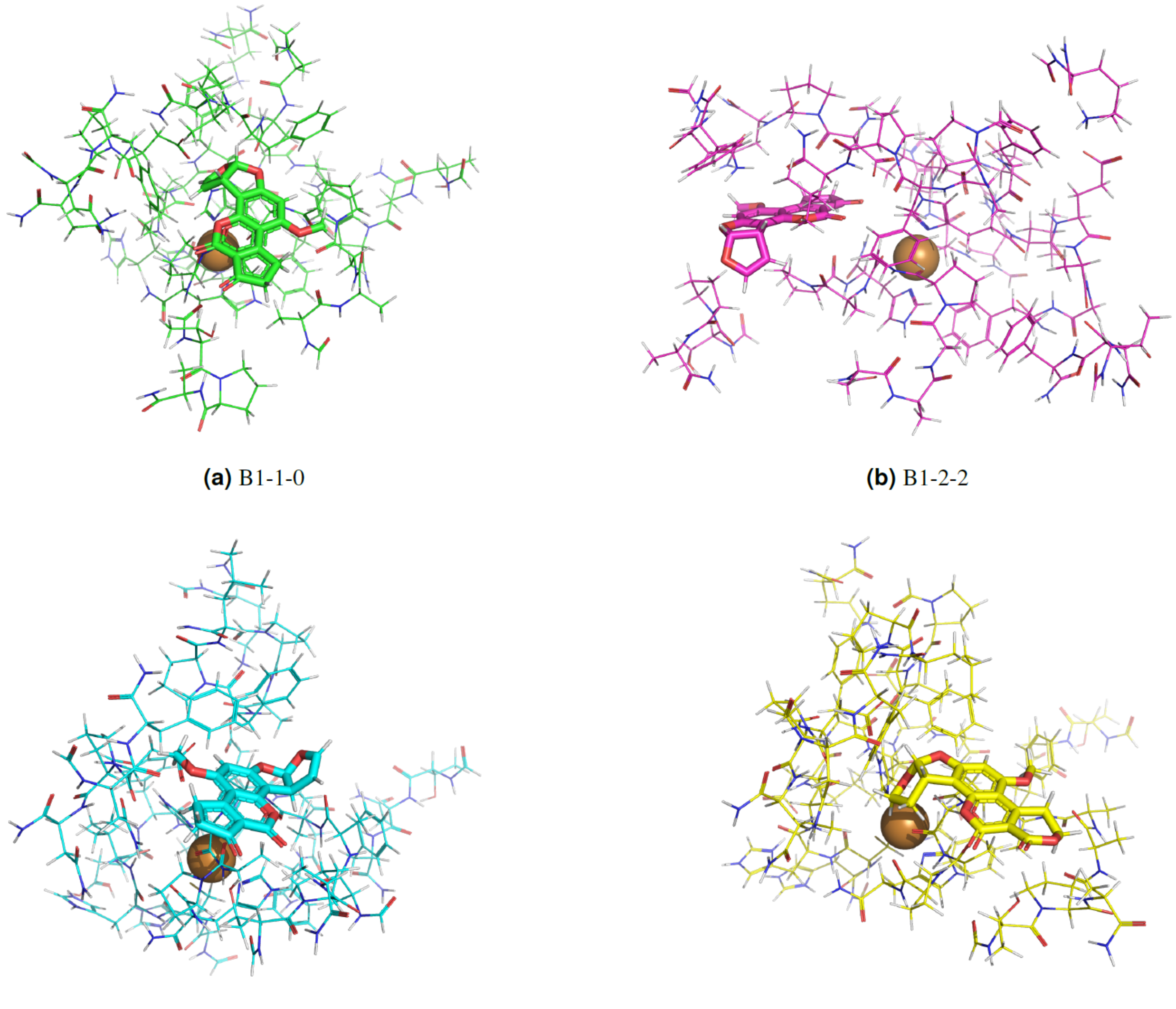

(a) B1-1-0

(b) B1-2-2

(c) B1-15-2

(d) G2-10-0

Supplementary Fig. S5. Active sites of four low energy binding poses for both AFB1 and AFG2 are shown.

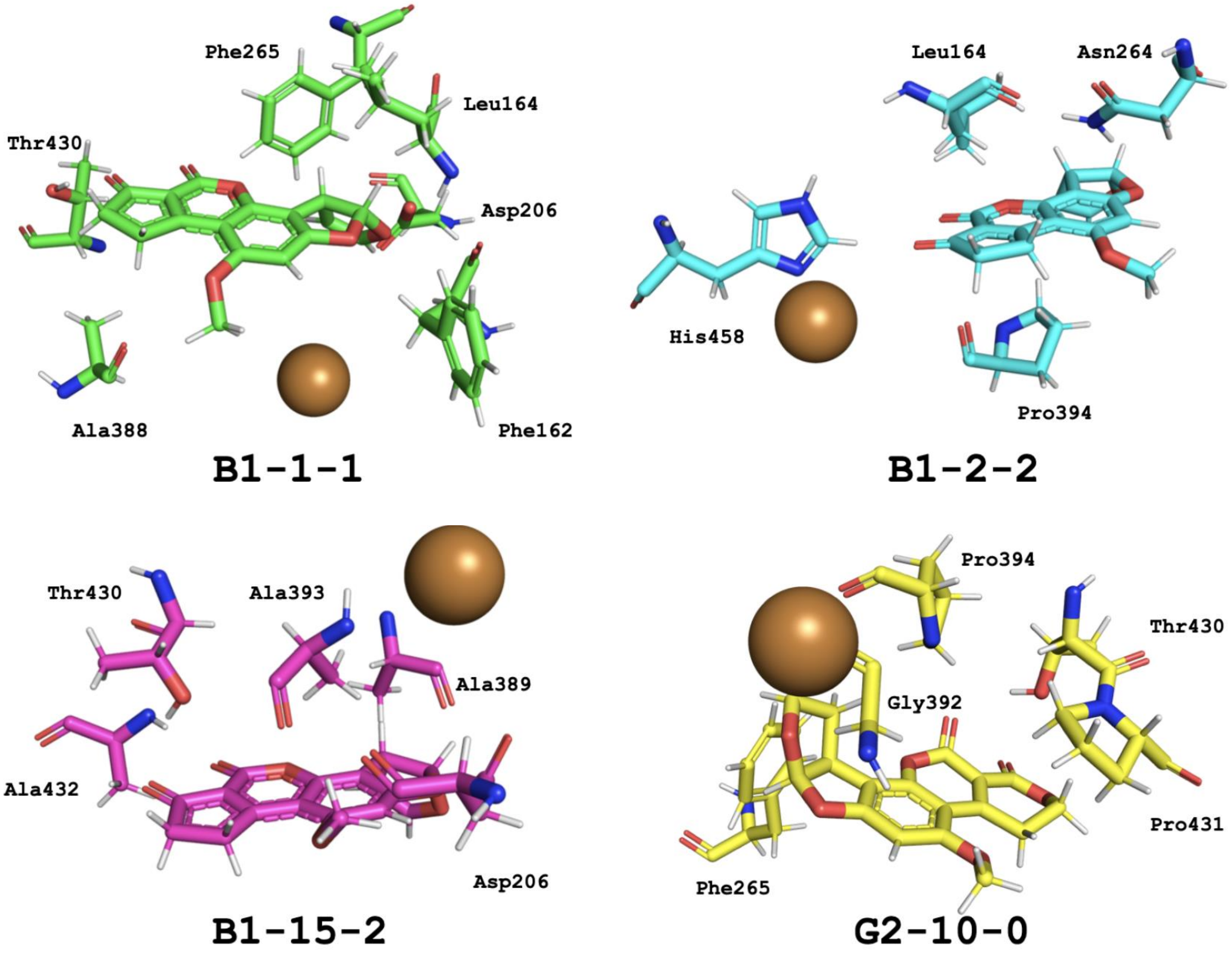

Supplementary Fig. S6. Active sites of low energy conformers of AFB1 and AFG2 with only strongly interacting residues (as determined by the FBO) are shown.

Docking Scores

Docking scores for different poses are provided in Table S1.

Table S1. Docking scores of the unique poses of the two toxins which are used as starting structure for molecular dynamics runs.

Toxin	Pose	GOLD Score
AFB_1	1	50.34
AFB_1	2	49.31
AFB_1	6	48.51
AFB_1	12	46.22
AFB_1	15	42.86
AFG_2	1	50.57
AFG_2	5	49.54
AFG_2	10	46.86
AFG_2	11	45.94
AFG_2	13	45.19
